# RSV‐Pre‐F Vaccination During Pregnancy and Neonatal Outcomes—A Systematic Review and Meta‐Analysis

**DOI:** 10.1002/iid3.70510

**Published:** 2026-09-09

**Authors:** Niko Leskinen, Marjut Haapanen, Ilari Kuitunen

**Affiliations:** ^1^ Institute of Clinical Medicine University of Eastern Finland Kuopio Finland; ^2^ Department of Gynecology and Obstetrics Mikkeli Central Hospital Mikkeli Finland; ^3^ Department of Pediatrics and Neonatology Kuopio University Hospital Kuopio Finland

## Abstract

**Objectives:**

Respiratory syncytial virus (RSV) is a major cause of infant morbidity and mortality worldwide. Recent preventive strategies include maternal vaccination and monoclonal antibodies. However, maternal RSV vaccination has raised safety concerns due to possible associations with preterm birth. This systematic review and meta‐analysis evaluated whether maternal RSV vaccination increases the risk of preterm birth and neonatal intensive care unit (NICU) admission.

**Methods:**

A systematic search of PubMed, Scopus, and Web of Science was conducted on August 1st, 2025. We used the following keywords in the search: RSV, vaccine, preterm, and neonate. Eligible studies included randomized controlled trials (RCTs) and observational studies assessing currently approved RSV‐PreF vaccines in pregnancy and reporting preterm birth (< 37 weeks) or NICU admission. Data extraction and risk of bias assessments (RoB 2.0, ROBINS‐I) were performed independently by two reviewers. Meta‐analyses were conducted using random‐effects models with risk ratios (RRs) as effect measures, and the certainty of evidence was graded using the GRADE framework.

**Results:**

Seven studies met inclusion criteria: three RCTs (*n* = 12,833 births) and four observational studies (*n* = 4245 births). In RCTs, RSV vaccination was associated with an increased risk of preterm birth (RR 1.26, 95% CI 1.08–1.46), while observational studies showed no association (RR 0.78, 95% CI 0.46–1.32). Pooled analysis revealed no overall association (RR 0.96, 95% CI 0.68–1.38). NICU admission risk was not significantly affected (RR 0.74, 95% CI 0.34–1.61). Certainty of evidence was rated low to very low.

**Conclusions:**

This meta‐analysis found no overall evidence linking maternal RSV vaccination to preterm birth, though RCT subgroup findings suggest a potential increased risk. Continued surveillance and long‐term safety monitoring are essential as global implementation expands.

**Trial Registration:** Open Science Framework (https://osf.io/3tfkn/).

## 1 Introduction

Respiratory syncytial virus (RSV) is the most common cause of hospitalization due to lower respiratory tract infections in infants [1]. With an estimate of 3.6 million hospital admissions and over one hundred thousand deaths annually among children aged 0–60 months, the global burden of RSV is substantial [2]. Prematurity, along with young age and underlying medical conditions, contribute most significantly to this global burden of RSV‐related hospitalizations [3]. In recent years, the development of monoclonal antibodies and maternal vaccination has played a crucial role in advancing RSV prevention [4].

Both nirsevimab, a monoclonal antibody, and maternal RSV vaccination are shown to reduce RSV‐related hospitalization, although there are differences between their efficacies favoring nirsevimab [5, 6]. A recent large cohort study directly comparing maternal RSVpreF vaccination with nirsevimab in preventing RSV‐related hospitalization in infants further supports this difference in effectiveness [7]. However, due to nirsevimab's current high cost, maternal vaccination is often used globally as the primary preventive strategy against RSV infection [8]. On the other hand, maternal vaccination has raised concerns, as some studies have linked it to an increased risk of premature birth leading to several trials being stopped [9, 10]. A previous rapid review with meta‐analysis included all tested maternal RSV vaccines reported an association with preterm birth [11]. As both vaccine and monoclonal antibodies remain widely in use, it is urgent to continue monitoring their efficacy and potential safety concerns.

Following these concerns, this study aimed to clarify whether maternal RSV vaccination increases the likelihood of premature birth and need for neonatal intensive care unit (NICU) admission. NICU admission was included as a second outcome because it captures a broader, more general indicator of neonatal safety following maternal RSV vaccination, beyond preterm birth alone.

## 2 Methods

We performed a systematic review and meta‐analysis and reported the findings according to the Preferred Reporting Items in Systematic Reviews and Meta‐Analysis (PRISMA) guidance [12]. The checklist is provided in the Supporting Materials.

### 2.1 Study Selection Process

We searched PubMed, Scopus, and Web of Science databases on August 1, 2025. The search used the following key terms: RSV OR respiratory syncytial virus AND vaccine AND preterm AND neonate. No language or date restrictions were applied. The complete search strategy is provided in the Supporting Materials. The search results were then uploaded to Covidence software (Veritas Healthcare Inc, Melbourne, Vic, Australia) for the screening process. Two authors (N. L. and I. K.) independently screened the abstracts and titles first and then full texts. Cases of disagreement were solved by the third author (M. H.).

### 2.2 Inclusion and Exclusion Criteria

We used the following PICOS in our inclusion and exclusion criteria. Patients needed to be pregnant individuals. The intervention was maternal administration of currently market approved RSV‐PreF vaccine. We excluded studies which were conducted with the nonmarket approved vaccines, as these trials were withheld prior due to increase in the preterm birth rate. Studies were eligible regardless of comparator, including placebo, no intervention, or active comparators. The reported outcome needed to be the rate of preterm births, and the preterm birth was defined as any birth occurring before gestational week 37 + 0. The study designs eligible for inclusion were randomized controlled trials (RCTs), prospective observational trials, retrospective observational trials. Other study designs were excluded.

### 2.3 Data Extraction

Data extraction was performed independently by two authors (N. L. and I. K.) to reduce potential extraction errors. The data extraction was performed to a predesigned data extraction template in Microsoft Excel.

### 2.4 Main Outcomes

We had two main outcomes in our review. The first outcome was the preterm birth rate, and the second main outcome was the need for NICU admission after birth. Birthweight and other neonatal morbidities were prespecified as additional outcomes and are reported descriptively where data were available. Due to insufficient and heterogeneous reporting across included studies, these outcomes were not subject to meta‐analysis.

### 2.5 Quality Assessment and Appraisal of Evidence Certainty

The risk of bias in the included studies was assessed independently by two authors (N. L. and I. K.) by the Cochrane risk of bias 2.0 tool for the RCTs and by ROBINS‐I (risk of bias in nonrandomized studies of interventions) for the observational studies [13, 14]. The risk of bias figures were generated by RobVis ShinyApp [15].

The certainty of evidence was assessed independently by the same two authors (N. L. and I. K.) according to the Core GRADE for both main outcomes [16].

### 2.6 Data Analysis

Data analysis was performed according to the guidance in the Cochrane handbook of systematic reviews latest version and analyses were conducted in RevMan statistical software. We pooled the studies together by inverse variance random effects meta‐analysis with Wald estimation. Risk ratios (RR) with 95% confidence intervals (CI) were used as effect estimates. We expected significant population heterogeneity and thus utilized a random‐effects model. A subgroup analysis was made separately for randomized trials and observational studies. Publication bias was analyzed by examining funnel plots if at least 10 studies reported the same outcome. Additional analyses were not performed.

## 3 Results

**Figure 1:**
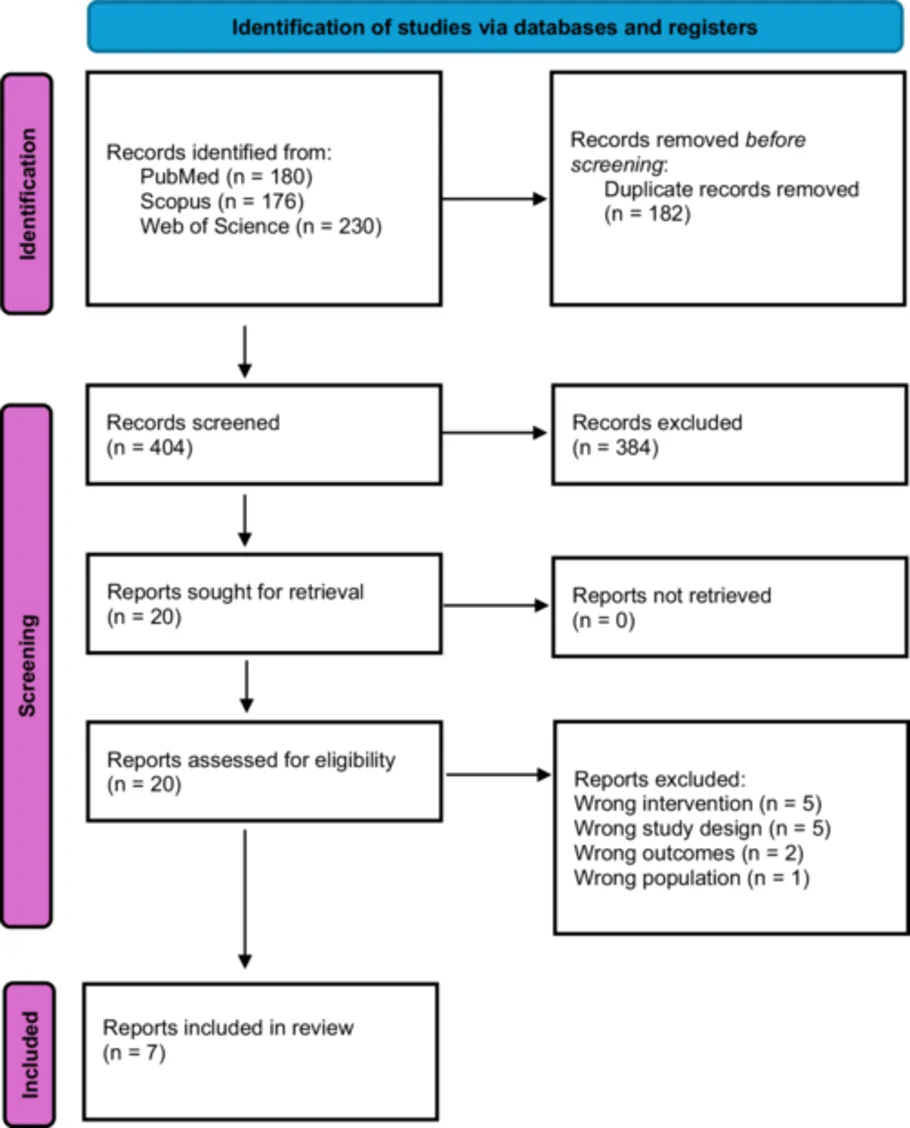
Flowchart of the study selection process.

**Figure 2:**
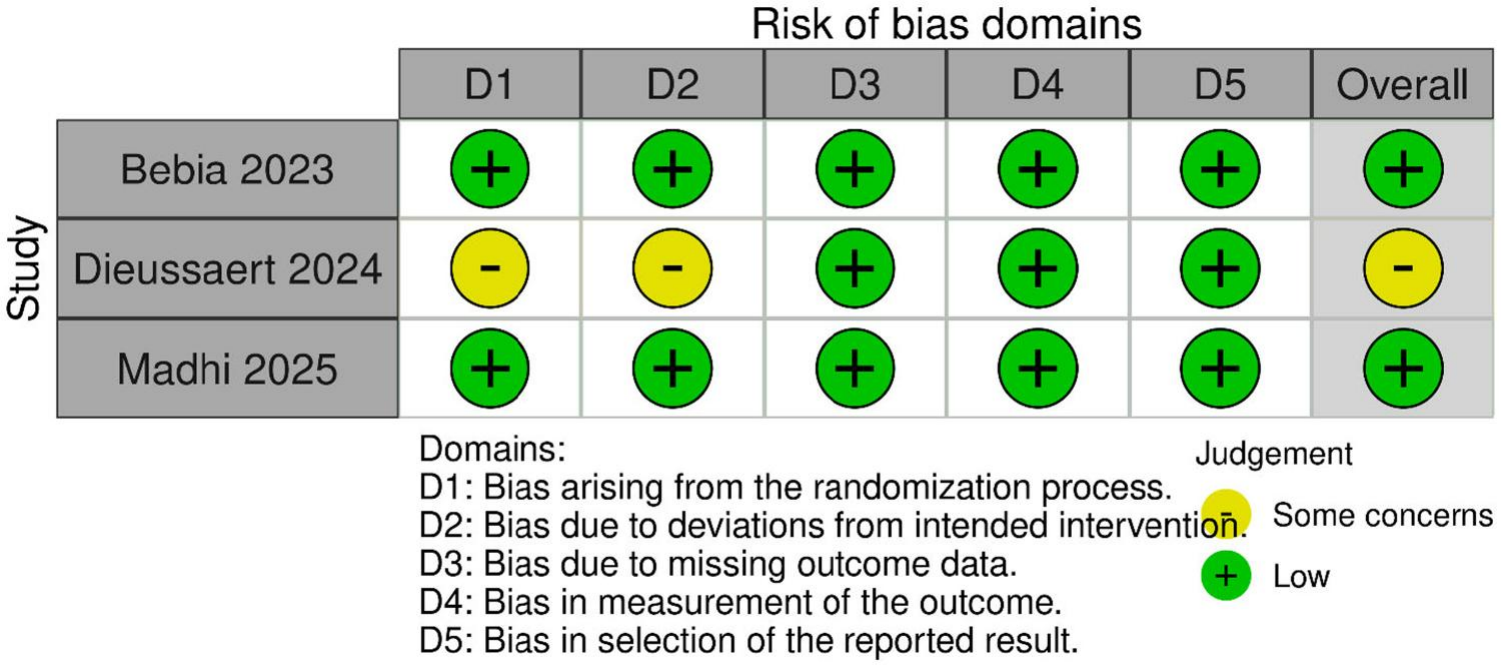
Risk of bias in randomized controlled trials assessed by the Cochrane RoB 2.0 tool. Both outcomes classified as subjective in the assessment.

**Figure 3:**
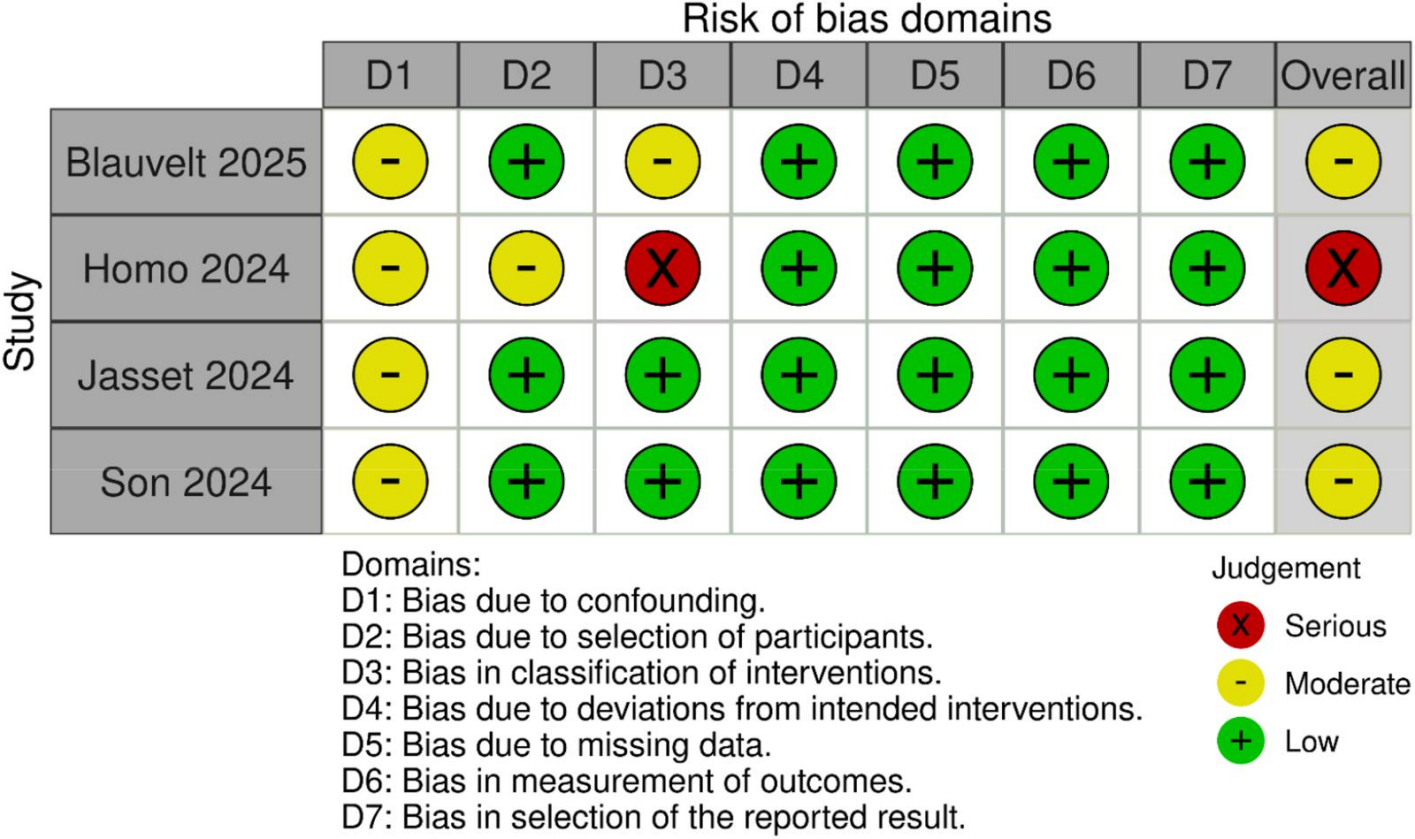
Risk of bias in observational studies assessed by the Cochrane ROBINS‐I tool. Both outcomes classified as subjective in the assessment.

Altogether, 404 studies were screened, of which 7 were included in the final main analysis [4, 9, 17, 18, 19, 20, 21] (Figure 1). Of the 7 included studies, 4 were observational studies with total of 4245 births and 3 were RCTs with a total of 12,833 births (Figure 1). The secondary analysis was based on the only two studies that reported NICU admissions [4, 19]. While all the observational studies were from the United States, all three of the RCTs were multinational. Gestational age at the time of the vaccination was typically between 24 and 36 weeks (Table 1). Birthweight was reported in four of the seven studies [4, 9, 18, 21] and was comparable between vaccine and control groups in each (means 3.2–3.34 kg vaccine vs 3.15–3.42 kg control); the remaining three studies [5, 17, 19] did not report birthweight. The risk of bias was low in two RCT's and had some concerns in one (Figure 2). In the observational studies, the risk of bias was moderate in three and serious in one (Figure 3).

**Table 1:** Characteristics of the included studies.

Study	Country	Design	Vaccination	Vaccination time	*N*, vaccine/control
Blauvelt et al. [4]	United States	Observational/retrospective	RSVPreF	GW 32 + 0 to 36 + 6	414/233
Bebia et al. [21]	Australia, Canada, Finland, France, New Zealand, Panama, South Africa, Spain, and the United States	RCT	RSVPreF3	GW 28 + 0 to 33 + 6	140/66
Dieussaert et al. [9]	24 countries	RCT	RSVPreF3‐Mat	GW 24 + 0 to 34 + 0	3557/1771
Homo et al. [17]	United States	Observational/retrospective	RSVPreF	GW 32 + 0 to 36 + 0	210/273
Jasset et al. [18]	United States	Observational/prospective	RSVPreF	GW 32 + 0 to 36 + 6	122/20
Madhi et al. [20]	18 countries	RCT	RSVPreF	GW 24 + 0 to 36 + 6	3656/3643
Son et al. [19]	United States	Observational/retrospective	RSVPreF	GW 32 + 0 to 36 + 6	1011/1962

**Figure 4:**
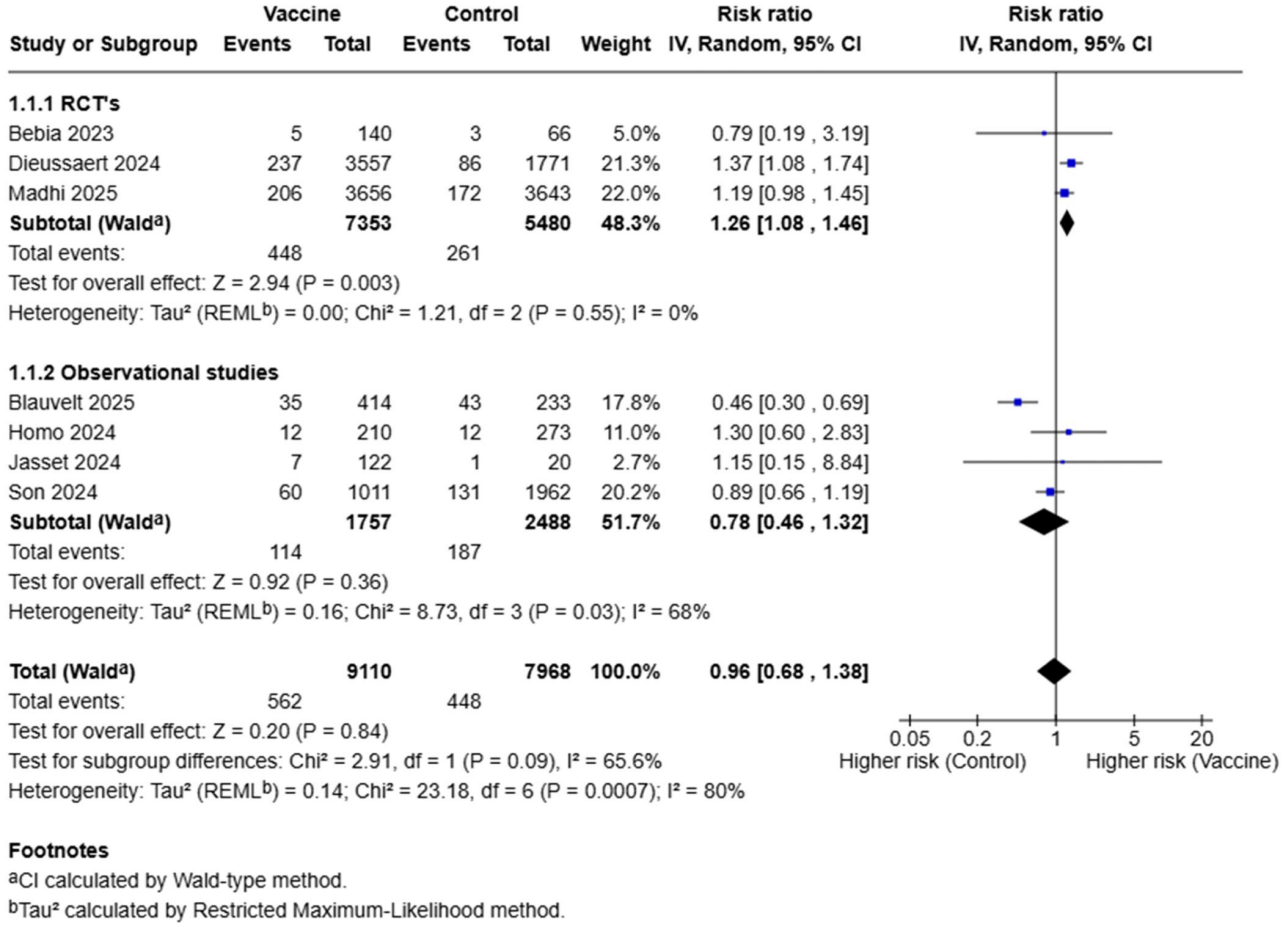
Forest plot of the preterm birth rate and subgroups based on study design.

**Figure 5:**
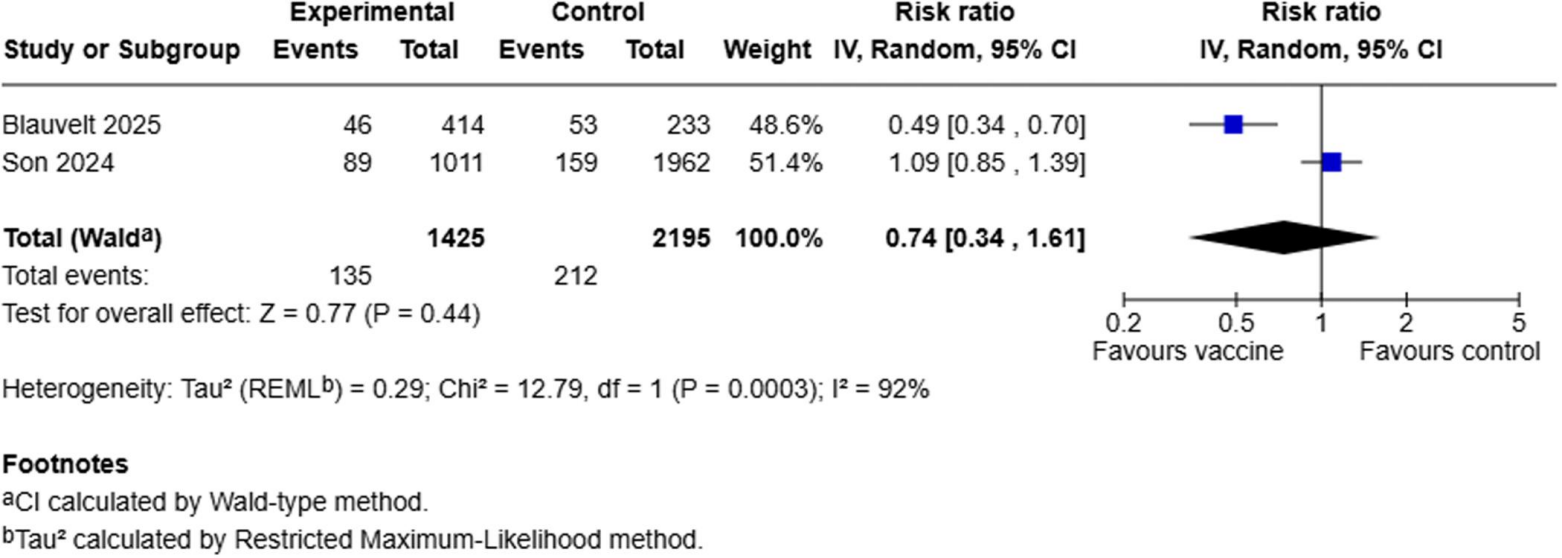
Forest plot for the need of neonatal intensive care unit admission.

In the randomized studies, a statistically significant increase in the risk of a preterm birth was observed with a relative risk of 1.26 (CI 1.08–1.46; *I* ^2^ 0%; Figure 4). However, the observational studies showed no evidence of an increased risk (RR 0.78, CI 0.46–1.32; *I* ^2^ 63%; Figure 4). The overall analysis of randomized and observational studies revealed no evidence of an association between RSV vaccination and preterm birth (RR 0.96, CI 0.68–1.38; *I* ^2^ 80%; Figure 4). We rated the certainty of evidence as low (Table 2). The secondary analysis did not reveal a statistically significant difference in NICU admission rates associated with RSV vaccination (RR 0.74, CI 0.34–1.61; *I* ^2^ 92%; Figure 5). We rated the certainty of evidence as very low (Table 2).

**Table 2:** The certainty of evidence for neonatal outcomes (preterm birth and need for neonatal intensive care unit admission) assessed according to GRADE.

Outcome	No. of participants (No. of studies)	Relative effect, RR (95% CI)	Absolute effect per 1000	GRADE
Preterm birth	19,178 (7)	RR 0.96 (0.68–1.38)	2 less preterm births (17 less to 22 more)	Low^a^
NICU admission	3620 (2)	RR 0.74 (0.34–1.61)	24 less NICU admissions (63 less to 57 more)	Very low^b^

*Note:* Reasons for downgrading

a Downgraded once due to risk of bias and once due to imprecision, as the CI includes both clear benefit and harm.

b Downgraded due to observational studies only providing data, imprecision, heterogeneity, and risk of bias.

## 4 Discussion

In this review of three RCTs and four observational studies, no statistically significant association was observed between RSV vaccination and preterm birth rates. However, a subgroup analysis of RCTs revealed a potential association between RSV vaccination and increased preterm birth rates, raising safety concerns. However, the discrepancy between randomized studies and observational studies is most likely explained by the different lower threshold gestational age limit. The randomized studies had lower gestational week limit of 24 or 28 for administration, whereas the observational studies had 32 weeks. Furthermore, all the observational studies came from the USA, whereas the randomized studies were multinational.

This is particularly important given that prematurity is the leading cause of death among children under five globally, and survivors often face increased odds of lifelong health complications [22]. Some previous studies have reported a higher incidence of premature births in the vaccine group, even leading to the discontinuation of one trial [9, 10, 20]. While the overall meta‐analysis did not confirm a statistically significant association, the subgroup findings warrant further investigation into vaccine safety.

Although preterm birth is a major risk factor for neonatal complications, including the need for intensive care, the NICU admission rate in this review was higher in the control group. However, the wide CI for the NICU admission outcome reflects substantial imprecision, attributable to the small number of contributing studies (*n* = 2) and the limited cumulative sample size available for this analysis. Firm conclusions regarding the effect of maternal RSV vaccination on NICU admission rates therefore cannot be drawn from the current evidence base. Thus, this topic still needs future studies.

Currently, the use of the RSV vaccine during pregnancy is approved in many countries, even though it is currently limited to a few countries such as the United Kingdom and the United States [23]. The Global Advisory Committee on Vaccine Safety has noted that, despite potential safety concerns—particularly regarding preterm birth—the benefits of maternal RSV vaccination outweighed the risks in 98% of simulations when administered between 27 and 36 weeks of gestation [24]. Based on these findings, recommendations on the use of maternal RSV vaccines were issued by the WHO Strategic Advisory Group of Experts (SAGE) in September 2024 and subsequently endorsed by WHO [25]. The real‐world efficacy of maternal RSV vaccination has already been demonstrated, as a study conducted in England and Scotland reported highly favorable outcomes from the 2024–25 RSV season [26].

Despite these recommendations and the proven efficacy of the vaccination, the potential use of hybrid RSV immunization strategies should still be considered, as the more expensive nirsevimab continues to offer superior protection, and the safety concerns regarding maternal vaccination remain [27].

The main strengths of this review are the conducted meta‐analysis and its timely contribution to the ongoing discourse on vaccine safety. The main limitation of this review is the limited availability of existing data, which reflects the early stage of research in this area. For example, neonatal morbidities were rarely reported, as the studies mainly focused on preterm birth and maternal safety outcomes. Due to the low number of included studies, publication bias could not be assessed properly. Additionally, this review was unable to monitor rare vaccine‐related side effects due to the lack of large‐scale nationwide studies. A further limitation is the search strategy: Web of Science was added as a database beyond the registered protocol, and ongoing trial registries (ClinicalTrials.gov, ICTRP) were not searched, which may have led to missed eligible studies.

## 5 Conclusions

We found no clear evidence of an association between maternal RSV vaccination and an increased risk of preterm birth overall. However, a statistically significant association was observed in the subgroup analysis of RCTs. Therefore, continued monitoring of potential side effects, particularly regarding preterm birth, is still essential as the global use of maternal RSV vaccination increases. Further studies should also aim to focus on other neonatal morbidities to further clarify the safety profile.

## Author Contributions

**Niko Leskinen:** writing – original draft, data curation, formal analysis. **Marjut Haapanen:** conceptualization, investigation, validation, data curation, writing – review and editing. **Ilari Kuitunen:** conceptualization, investigation, writing – review and editing, visualization, validation, methodology, software, formal analysis, project administration, supervision, resources. All authors approved the final version to be submitted.

## Ethics Statement

None needed due to systematic review study design.

## Consent

None needed due to systematic review study design.

## Conflicts of Interest

The authors declare no conflicts of interest.

## Supporting information


Supporting File

## Data Availability

All data generated during the review process are available from the corresponding author.

## References

[1] E. Thomas , J. M. Mattila , P. Lehtinen , T. Vuorinen , M. Waris , and T. Heikkinen , “Burden of Respiratory Syncytial Virus Infection During the First Year of Life,” Journal of Infectious Diseases 223, no. 5 (March 2021): 811–817, 10.1093/infdis/jiaa754. 33350450

[2] T. Shi , D. A. McAllister , K. L. O'Brien , et al., “Global, Regional, and National Disease Burden Estimates of Acute Lower Respiratory Infections Due to Respiratory Syncytial Virus in Young Children in 2015: A Systematic Review and Modelling Study,” Lancet 390, no. 10098 (September 2017): 946–958, 10.1016/S0140-6736(17)30938-8. 28689664 PMC5592248

[3] X. Wang , Y. Li , T. Shi , et al., “Global Disease Burden of and Risk Factors for Acute Lower Respiratory Infections Caused by Respiratory Syncytial Virus in Preterm Infants and Young Children in 2019: A Systematic Review and Meta‐Analysis of Aggregated and Individual Participant Data,” Lancet 403, no. 10433 (March 2024): 1241–1253, 10.1016/S0140-6736(24)00138-7. 38367641

[4] C. A. Blauvelt , M. Zeme , A. Natarajan , et al., “Respiratory Syncytial Virus Vaccine and Nirsevimab Uptake Among Pregnant People and Their Neonates,” JAMA Network Open 8, no. 2 (February 2025): e2460735, 10.1001/jamanetworkopen.2024.60735. 39969879 PMC11840647

[5] B. Kampmann , S. A. Madhi , I. Munjal , et al., “Bivalent Prefusion F Vaccine in Pregnancy to Prevent RSV Illness in Infants,” New England Journal of Medicine 388, no. 16 (April 2023): 1451–1464, 10.1056/NEJMoa2216480. 37018474

[6] P. I. Babawale , I. Martínez‐Espinoza , A. M. Mitchell , and A. Guerrero‐Plata , “Preventing RSV Infection in Children: Current Passive Immunizations and Vaccine Development,” Pathogens 14, no. 2 (February 2025): 104, 10.3390/pathogens14020104. 40005481 PMC11858734

[7] M. J. Jabagi , M. Bertrand , A. Gabet , E. Kolla , V. Olié , and M. Zureik , “Nirsevimab vs RSVpreF Vaccine for Respiratory Syncytial Virus‐Related Hospitalization in Newborns,” Journal of the American Medical Association 335, no. 9 (March 2026): 787–798, 10.1001/jama.2025.24082. 41428474 PMC12723599

[8] A. C. Langedijk , F. van den Dungen , L. Harteveld , et al., “Cost‐Effectiveness of Immunization Strategies to Protect Infants Against Respiratory Syncytial Virus in the Netherlands,” Human Vaccines & Immunotherapeutics 21, no. 1 (December 2025): 2521912, 10.1080/21645515.2025.2521912. 40561047 PMC12934181

[9] I. Dieussaert , J. Hyung Kim , S. Luik , et al., “RSV Prefusion F Protein–Based Maternal Vaccine — Preterm Birth and Other Outcomes,” New England Journal of Medicine 390, no. 11 (March 2024): 1009–1021, 10.1056/NEJMoa2305478. 38477988

[10] H. Boytchev , “Maternal RSV Vaccine: Further Analysis Is Urged on Preterm Births,” BMJ 381 (May 2023): p1021, 10.1136/bmj.p1021. 37164373

[11] I. Kuitunen and M. Haapanen , “Respiratory Syncytial Virus Vaccination Is Associated With Increased Odds of Preterm Birth,” Acta Paediatrica (January 2025), 10.1111/apa.17595. PMC13659349 39831688

[12] A. Liberati , D. G. Altman , J. Tetzlaff , et al., “The PRISMA Statement for Reporting Systematic Reviews and Meta‐Analyses of Studies That Evaluate Healthcare Interventions: Explanation and Elaboration,” BMJ 339 (July 2009): b2700, 10.1136/bmj.b2700. 19622552 PMC2714672

[13] J. A. Sterne , M. A. Hernán , B. C. Reeves , et al., “ROBINS‐I: A Tool for Assessing Risk of Bias in Non‐Randomised Studies of Interventions,” BMJ 355 (October 2016): i4919, 10.1136/bmj.i4919. 27733354 PMC5062054

[14] J. A. C. Sterne , J. Savović , M. J. Page , et al., “RoB 2: A Revised Tool for Assessing Risk of Bias in Randomised Trials,” BMJ 366 (August 2019): l4898, 10.1136/bmj.l4898. 31462531

[15] L. A. McGuinness and J. P. T. Higgins , “Risk‐of‐Bias VISualization (Robvis): An R Package and Shiny Web App for Visualizing Risk‐of‐Bias Assessments,” Research Synthesis Methods 12, no. 1 (January 2021): 55–61, 10.1002/jrsm.1411. 32336025

[16] G. Guyatt , T. Agoritsas , R. Brignardello‐Petersen , et al., “Core GRADE 1: Overview of the Core GRADE Approach,” BMJ 389 (April 2025): e081903, 10.1136/bmj-2024-081903. 40262844

[17] R. L. Homo , A. Groberg , M. Donahue , D. Halverson , A. Wooten , and A. Ponnapakkam , “High Uptake of Respiratory Syncitial Virus Prevention for Neonates in a Military Treatment Facility,” Journal of Pediatrics 273 (October 2024): 114144, 10.1016/j.jpeds.2024.114144. 38876155

[18] M. O. J. Jasset , P. A. L. Zapana , Z. Bahadir , et al., Longer Interval Between Maternal RSV Vaccination and Birth Increases Placental Transfer Efficiency. Cold Spring Harbor Laboratory, 2024), 10.1101/2024.07.14.24310390.

[19] M. Son , L. E. Riley , A. P. Staniczenko , et al., “Nonadjuvanted Bivalent Respiratory Syncytial Virus Vaccination and Perinatal Outcomes,” JAMA Network Open 7, no. 7 (July 2024): e2419268, 10.1001/jamanetworkopen.2024.19268. 38976271 PMC11231799

[20] S. A. Madhi , B. Kampmann , E. A. F. Simões , et al., “Preterm Birth Frequency and Associated Outcomes From the MATISSE (Maternal Immunization Study for Safety and Efficacy) Maternal Trial of the Bivalent Respiratory Syncytial Virus Prefusion F Protein Vaccine,” Obstetrics and Gynecology 145, no. 2 (2025): 147–156, 10.1097/AOG.0000000000005817. 39746206 PMC11731028

[21] Z. Bebia , O. Reyes , R. Jeanfreau , et al., “Safety and Immunogenicity of an Investigational Respiratory Syncytial Virus Vaccine (RSVPreF3) in Mothers and Their Infants: A Phase 2 Randomized Trial,” Journal of Infectious Diseases 228, no. 3 (August 2023): 299–310, 10.1093/infdis/jiad024. 36722147 PMC10420396

[22] “150 Million Babies Born Preterm in the Last Decade,” cited August 7, 2025, https://www.unicef.org/press-releases/150-million-babies-born-preterm-last-decade.

[23] “Respiratory Syncytial Virus (RSV),” cited August 8, 2025, https://www.who.int/groups/global-advisory-committee-on-vaccine-safety/topics/rsv.

[24] “WER10031‐eng‐fre.pdf,” cited August 8, 2025, https://iris.who.int/bitstream/handle/10665/382141/WER10031-eng-fre.pdf.

[25] “WER10022‐eng‐fre.pdf,” cited August 8, 2025, https://iris.who.int/bitstream/handle/10665/381539/WER10022-eng-fre.pdf?sequence=1.

[26] T. C. Williams , R. Marlow , S. Cunningham , et al., “Bivalent Prefusion F Vaccination in Pregnancy and Respiratory Syncytial Virus Hospitalisation in Infants in the UK: Results of a Multicentre, Test‐Negative, Case‐Control Study,” Lancet Child & Adolescent Health 9, no. 9 (September 2025): 655–662, 10.1016/S2352-4642(25)00155-5. 40690922

[27] G. B. Gebretekle , M. W. Yeung , R. Ximenes , et al., “Cost‐Effectiveness of RSVpreF Vaccine and Nirsevimab for the Prevention of Respiratory Syncytial Virus Disease in Canadian Infants,” Vaccine 42, no. 21 (August 2024): 126164, 10.1016/j.vaccine.2024.126164. 39079810

